# Merging functional and structural properties of the monkey auditory cortex

**DOI:** 10.3389/fnins.2014.00198

**Published:** 2014-07-21

**Authors:** Olivier Joly, Simon Baumann, Fabien Balezeau, Alexander Thiele, Timothy D. Griffiths

**Affiliations:** Auditory Group, Institute of Neuroscience, Newcastle UniversityNewcastle Upon Tyne, UK

**Keywords:** auditory cortex, monkey, tonotopy, phase-encoded design, cortical surface

## Abstract

Recent neuroimaging studies in primates aim to define the functional properties of auditory cortical areas, especially areas beyond A1, in order to further our understanding of the auditory cortical organization. Precise mapping of functional magnetic resonance imaging (fMRI) results and interpretation of their localizations among all the small auditory subfields remains challenging. To facilitate this mapping, we combined here information from cortical folding, micro-anatomy, surface-based atlas and tonotopic mapping. We used for the first time, phase-encoded fMRI design for mapping the monkey tonotopic organization. From posterior to anterior, we found a high-low-high progression of frequency preference on the superior temporal plane. We show a faithful representation of the fMRI results on a locally flattened surface of the superior temporal plane. In a tentative scheme to delineate core versus belt regions which share similar tonotopic organizations we used the ratio of T1-weighted and T2-weighted MR images as a measure of cortical myelination. Our results, presented along a co-registered surface-based atlas, can be interpreted in terms of a current model of the monkey auditory cortex.

## Introduction

**Figure 1:**
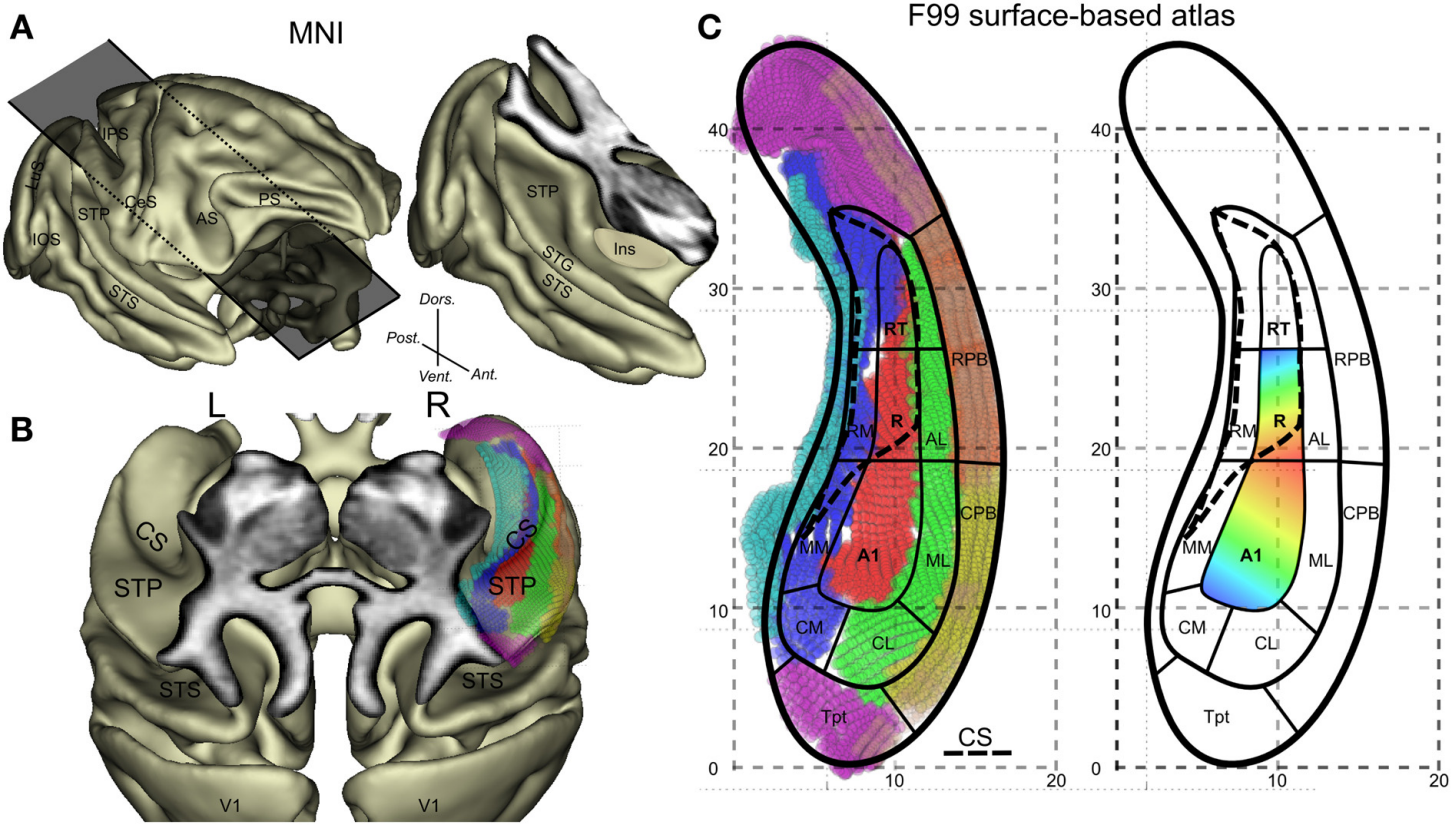
**General anatomical description of the monkey auditory cortex and its subdivisions. (A)** Lateral view of the 3D cortical surface reconstructed from the MNI-space template and illustration of an oblique cut above the temporal lobe. **(B)** Top oblique view on the surface of the superior temporal plane with identification of the circular sulcus (CS). The overlay of the F99 surface based atlas and its auditory subfields onto the right lateral sulcus are also shown. **(C)** Detailed subdivisions of the auditory subfields with vertex in different colors and schematic outlines and typical tonotopic organization in A1 and R. High(blue)-Low(red)-High progression of frequency tuning along a postero-anterior axis. Other abbreviations: IPS, intraparietal sulcus; CeS, central sulcus; AS, arcuate sulcus; PS, principal sulcus; IOS, inferior occipital sulcus; LuS, lunate sulcus; STS, superior temporal sulcus; Ins, insula; CM, caudomedial auditory belt; CL, caudal lateral auditory belt; ML, middle lateral auditory belt; MM, middle medial auditory belt; RM, rostromedial region; AL, anterior lateral auditory belt; CPB, caudal auditory parabelt; RPB, rostral auditory parabelt; Tpt, temporoparietal area.

The auditory cortex is located in the temporal lobe of the primate brain and in macaque monkeys it lies mainly on the superior temporal plane (Figures 1A,B). The monkey auditory cortex can be divided into three core regions surrounded by seven or eight belt areas which project mainly to parabelt regions on the convexity of the superior temporal gyrus (Hackett, 2011). The current model of organization of the monkey auditory cortex consists of 12 regions which are defined on the basis of architectonic boundaries and connections (Jones et al., 1995; Hackett et al., 1998). Neurophysiological studies and more recently functional magnetic resonance imaging studies (fMRI) studies (Petkov et al., 2006, 2008; Baumann et al., 2010; Tanji et al., 2010; Joly et al., 2012b) aimed to improve our understanding of the functional properties of these auditory subfields. However, the attribution of auditory cortical subfields in fMRI studies can be problematic, because of imaging issues (e.g., EPI distortion and partial volume effects) and problems with the distinction between adjacent core and narrow belt areas that have the same tonotopic preference.

Imaging issues related to field definition using fMRI are manifold: (1) Spatial resolution is currently between 1 and 2 mm which is about the width of medial belt regions. (2) Echo-planar images (T2^*^-weighted fMRI) typically show geometric distortions which are only partially corrected contributing to misalignment of fMRI results with anatomical images. (3) Voxel-based representation of the auditory cortex using axial slices or oblique slices aligned with the posterior part of the superior temporal plane cannot represent faithfully the anterior auditory cortex which is folded, in particular at the level of the circular sulcus (Figures 1B,C).

Tonotopy is a basic organizing principle along the auditory pathway including the primary auditory cortex. In macaques, many neurons in the superior temporal plane respond to auditory tones and show a tuning for specific stimulation frequencies. The auditory core neurons in particular, which receive direct input from the strongly tonotopically organized ventral division of the medial geniculate complex, show an organization of characteristic frequencies, which progresses mainly along a posterior-anterior axis (Figure 1C). Reversals in this progression define functionally the borders of 3 core regions: from posterior to anterior these are A1, R, and RT (Figure 1C) (Merzenich and Brugge, 1973; Morel et al., 1993). Tonotopic maps in the core region form a continuum with frequency preference found in the surrounding belt areas (see illustration in Figure 1 in Hackett et al., 2001). This shared frequency tuning between core and adjacent belt regions means that this property cannot be used to functionally define a border between core and belt regions. Other functional properties have been suggested to distinguish core and belt such as selectivity of frequency tuning (Moerel et al., 2012), response latency and preference for noise over pure tones in belt areas (Recanzone, 2000; Rauschecker and Tian, 2004). However, neural response latency is difficult to assess with fMRI because of its temporal resolution (heamodynamic filter) and the latency difference between core and belt is small (Camalier et al., 2012). Anatomical consideration provides a more direct measure of core. At the microscopic level, a major anatomical characteristic of the core (mainly A1 and R) is that it shows heavy staining for parvalbumin, acetylcholinesterase, cytochrome-oxidase, and myelin as compared to belt regions (Hackett, 2011). At the macroscopic level, anatomical landmarks can largely predict the functional borders in humans, where the utility of the transverse temporal gyrus has been emphasized (Da Costa et al., 2011) although micro-anatomy (cyto-architectural borders) does not always follow macro-anatomical landmarks (Morosan et al., 2001). Rhesus monkeys do not have transverse temporal gyrus but do have other anatomical features that we assess here.

Here, we present a set of data and analyses integrating information from 4 different sources: (1) tonotopic mapping based on phase-encoding fMRI design known to be efficient, producing relatively robust maps from relatively short scanning time (Da Costa et al., 2011; Engel, 2012). (2) Macro-anatomical features related to the curvature of the cortical surface. (3) Mapping of anatomical properties of the auditory cortex using an index derived from the ratio of T1 over T2 weighted MR images (Glasser and Van Essen, 2011). (4) A surface-based anatomical atlas. This has allowed us to produce a detailed map of areal organization on the 3-dimensional superior temporal plane based on fMRI and T1 and T2 maps and a current model of organization (Hackett et al., 1998; Hackett, 2011; Baumann et al., 2013). Comparison of the map with macroscopic anatomy suggests that the low-frequency representation at the border between A1 and R is associated with the posterior end of the circular sulcus.

## Materials and methods

### Subjects

Two male adult rhesus monkeys (*Macaca mulatta*), 10 and 5 years of age, weighing 18 and 11 kg, participated in the experiment. The animals, M1 and M2, had previous exposure to experimental auditory stimuli. Before the scanning sessions, monkeys were trained to perform a visual fixation task with the head of the animal rigidly positioned with a head holder attached to a cranial implant (see Thiele et al., 2006 for details regarding details and surgical procedures). The visual fixation task was used to equalize as much as possible attention across runs and more importantly to minimize stress and body movement during scanning. All experiments were carried out in accordance with the European Communities Council Directive RL 2010/63/EC, the US National Institutes of Health Guidelines for the Care and Use of Animals for Experimental Procedures and the UK Animals Scientific Procedures Act and were performed with great care to ensure the well-being of the animals.

### Stimuli

**Figure 2:**
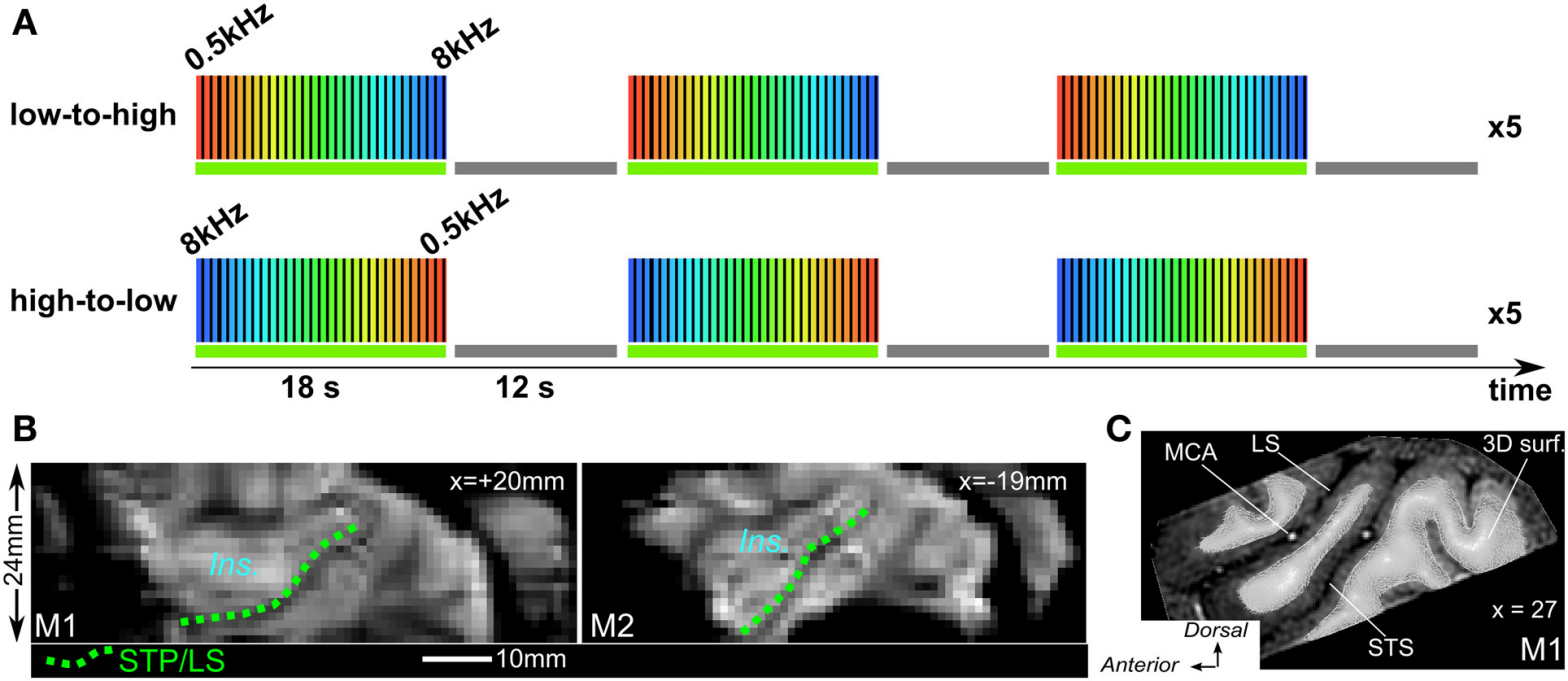
**Phase-encoded fMRI design and mean image of times series. (A)** Both run types were alternated with either low-to-high (top) or hig-to-low frequency progression and “ON” block was always followed by a 12 s. “OFF” block (baseline). **(B)** Sagittal view from mean image of time series for both M1 and M2. Dashed green lines illustrate the lower bank of the lateral sulcus or STP to which the 3D surfaces are adjusted. *Ins.:* insula cortex. **(C)** Sagittal view from anatomical MRI with 3D mesh surface derived from the segmentation of the white matter (M1) MCA, middle cerebral artery.

Sound stimuli were generated at the beginning of each functional run. The stimuli were computed with a sampling rate of 44.1 kHz using a custom-made python-based program—*PrimatePy,* which mainly relies on Psychopy (Peirce, 2007), a psychophysics package (www.psychopy.org/). *PrimatePy* also uses other python libraries for the generation of sound arrays and for the control of the multi-threading architecture. Stimuli were pure tone bursts and were presented in either low-to-high or high-to-low progression of frequencies (Figure 2A). Frequencies were 500, 707, 1000, 1414, 2000, 2828, 4000, 5657, and 8000 Hz (half-octave steps). Tone bursts were either 50 ms or 200 ms in duration (inter-stimulus interval 50 ms) and were alternated in pseudo-randomized order during the 2 s block. Pure tone bursts of each frequency were presented in 2 s blocks in succession until all 9 frequencies had been presented. This 18 s low-to-high progression was followed by a 12 s silent pause, and this 30 s cycle was presented 15 times. A run lasted for 8 min and the two run types with either low-to-high or high-to-low progression (Figure 2A), were alternated. Stimuli were delivered through MR-compatible insert earphones (sensimetrics, Model S14, www.sens.com) at about 75–80 dB sound pressure level (SPL). Scan noise was attenuated by the insert earphones and by dense foam padding around the ears.

### Behavioral task

The animal performed the visual fixation task during the acquisition of a full time series (8 min). Each time series was followed by a break of about 1 min. The eye position was monitored at 60 Hz with a tracking (camera-based with Infra-Red illumination) of the pupil using iView software (SMI, www.smivision.com, Teltow, Germany). The eye position, *X* and *Y* coordinates, was communicated to PrimatePy via a UDP/IP socket. The task was as follow: a fixation target (a small red square) appeared on the center of the screen, when the eye trace entered within a fixation window (about 2–3 visual degree centered onto the target) a timer started and the fixation target turned green. A continuous visual fixation (no saccades) of a randomly defined duration of 2–2.5 s. was immediately followed by the delivery of a juice reward using a gravity-fed dispenser. The reward was controlled by PrimatePy via a data acquisition USB device LabJack (U3-LV, http://labjack.com/).

### Data acquisition—magnetic resonance imaging

Magnetic resonance images were acquired at 4.7 Tesla with an actively shielded vertical scanner (Bruker Biospec 47/60 VAS, inner-bore width of 38 cm, Bruker GA-385 gradient system) dedicated to imaging non-human primates. Shimming was performed with the FASTMAP (Gruetter, 1993) algorithm which uses projections through a predefined volume to measure B0 inhomogeneity and applies first and second order corrections. Functional MRI measurements by blood oxygen level-dependent (BOLD) contrast consisted of single-shot gradient-echo echo-planar imaging (GE-EPI) sequences with the following parameters: *TR* = 1400 ms, *TE* = 21 ms, 90° flip angle, Receiver Bandwidth (BW) = 138 kHz, matrix size 92 × 92, FOV 110 × 110 mm, in-plane resolution 1.2 × 1.2 mm, slice thickness = 1.2 mm, yielding to 1.72 mm^3^ voxels. Functional time series lasted 8 min and consisted of a continuous acquisition of 343 volumes (plus 2 dummy scans) with 20 axial interleaved slices (ascending order, no gaps) acquired with parallel imaging with 2-fold GRAPPA acceleration using 8-channel array receive coil. The RF transmission was done with the Bruker birdcage volume coil in transmit mode. From the scanner, a TTL pulse signal was triggered at the start of every volume and sent out to PrimatePy via the LabJack for synchronization purposes. In total, a number of 23 runs were acquired (M1:15, M2:8) which represents 23*343 = 7889 EPIs. Based on the behavior (amount of body motion), only a subset of 16 runs entered into the analyses (M1:8, M2:8).

Anatomical MR images consisted of 2 sequences, T1-weighted (T1w) and T2 weighted (T2w) images. The T1w images consisted of a 2D magnetization-prepared rapid gradient-echo (MPRAGE) sequence with a 130° preparation pulse, *TR* = 2100 ms, *TE* = 7 ms, *TI* = 800 ms, 27° flip angle, Receiver Bandwidth = 30 KHz. The T2w images consisted of a 2D Rapid Acquisition with Relaxation Enhancement (RARE) sequence with *TR* = 6500 ms, *TE* = 14 ms, Effective *TE* = 56 ms, *BW* = 50 kHz, RARE factor 8. The geometry was the same for both T1w and T2w images: matrix 166 × 166, FOV 100 × 100 mm, slices thickness 0.6 mm, and 54 axial slices. Because of time constraints, those anatomical scans were acquired during separate scanning sessions but with the same visual fixation task to minimize body motion and stress and to control the animal's behavior.

### Data analyses

MR images were first converted from Bruker file format into 3D (anatomical data) or 4D (*x, y, z, t* functional data) minc file format (.mnc) using the Perl script pvconv.pl available online (http://pvconv.sourceforge.net/) and next from minc to nifti format using the minc tools.

#### Structural images

Structural images were resampled at 0.25 mm isotropic voxels with 7th order B-spline interpolation method and reoriented to MNI space (alignment of posterior and anterior commissures). The resampling allows a smoother definition of the cortical surface: interface between gray and white matter. The ratio between T1w and T2w structural images was used to segment the brain as it increases the contrast between white and gray matter and reduces biases from B0 inhomogeneities and from the receiver sensitivity profile (Glasser and Van Essen, 2011). Next, semi-automatic segmentation (Yushkevich et al., 2006) of the white matter was performed using ITKsnap (http://www.itksnap.org). The binary image (after dilation of 0.25 mm) was used to generate a 3D triangulated mesh (Figure 2C) including smoothing and decimation to reduce the number of vertices using BrainVisa suite (http://brainvisa.info) and a selection of the sub-surface corresponding to the superior temporal plane (lower bank of the lateral sulcus) was saved into the GIFTI (www.nitrc.org/projects/gifti/) file format. The STP surface area was about 350 mm^2^ in M1 and about 280 mm^2^ in M2. The atlas surface from the macaque F99 atlas which is available in Caret software (Van Essen et al., 2001) was co-registered with our surfaces using ICP (Iterative Closest Point) registration (affine registration) as implemented in vtk (www.vtk.org). The benefit of this local surface-based registration is to enable the comparison of our functional MRI results with the anatomical atlas (Markov et al., 2011) which uses the well-established nomenclature and subdivisions of the auditory cortex defined earlier (Hackett et al., 1998; Kaas and Hackett, 1998). To improve the visibility of the cortical surface we flattened the superior temporal plane. As this flattening process is only applied to a local part of the cortical surface it does not require full brain inflation and spherical coordinates transformation. Instead it consist of a two-step procedure: (1) computation of the weighted adjacency matrix that reflects position of the vertices (Dijkstra's algorithm). (2) Computation of the multidimensional scaling (MDS) of the adjacency matrix (Joly et al., 2009).

T1w/T2w ratio images were sampled across cortical depth: along the normal of the surface at each vertex. Sampling along the normal was initiated on the slightly dilated gray/white matter surface. At each vertex, along the normal T1w/T2w, values exceeding ±1 SD of all values were excluded: this had the effect of removing voxels that contained significant blood vessel signal with very high T1w/T2w values or CSF signal with very low values (Glasser and Van Essen, 2011). Finally, maps were smoothed across the surface using a gaussian average weighted by geodesic distance (FWHM = 1 mm) that reduced high spatial frequency information, which appeared to be mostly noise.

#### Functional images

Raw fMRI data entered into a preprocessing stage using Statistical Parametric Mapping (SPM8) software (*www.fil.ion.ucl.ac.uk/spm/*), including slice timing correction and rigid body motion correction. The fMRI data were also spatially smoothed with a Gaussian kernel (FWHM = 1.5 mm). Voxel-based analyses were performed in SPM and consisted of the estimation of a general linear model (GLM) with a block design of alternating (18 s) ON and (12 s) OFF blocks and including the motion parameters. The quality of the co-registration of the 3D LS surface with the functional time series was visually verified using mean time series (Figure 2B). Projection of the functional volumetric data onto the cortical surface (Figure 2C) was then performed with Caret command line (interpolated voxel method) and resulted into surface-based (texture) time-series. The resulting time-series were further processed using python scripts (nitime and nibabel python libraries). Times series entered a filter with an infinite impulse response (IIR) function to remove fluctuations below 0.02 and above 0.1 Hz. The filtered times series of each vertex was then normalized as percentage of signal change relative to the mean signal of that vertex. For each vertex, cross-correlation between time-series from both run types was computed and time delay between the two signals (argument of the maximum correlation) revealed the preferred frequency. Maps of frequency preference were generated for the computation at each vertex and using an inclusive mask of correlation values above 0.2.

## Results

### Sound activations

**Figure 3:**
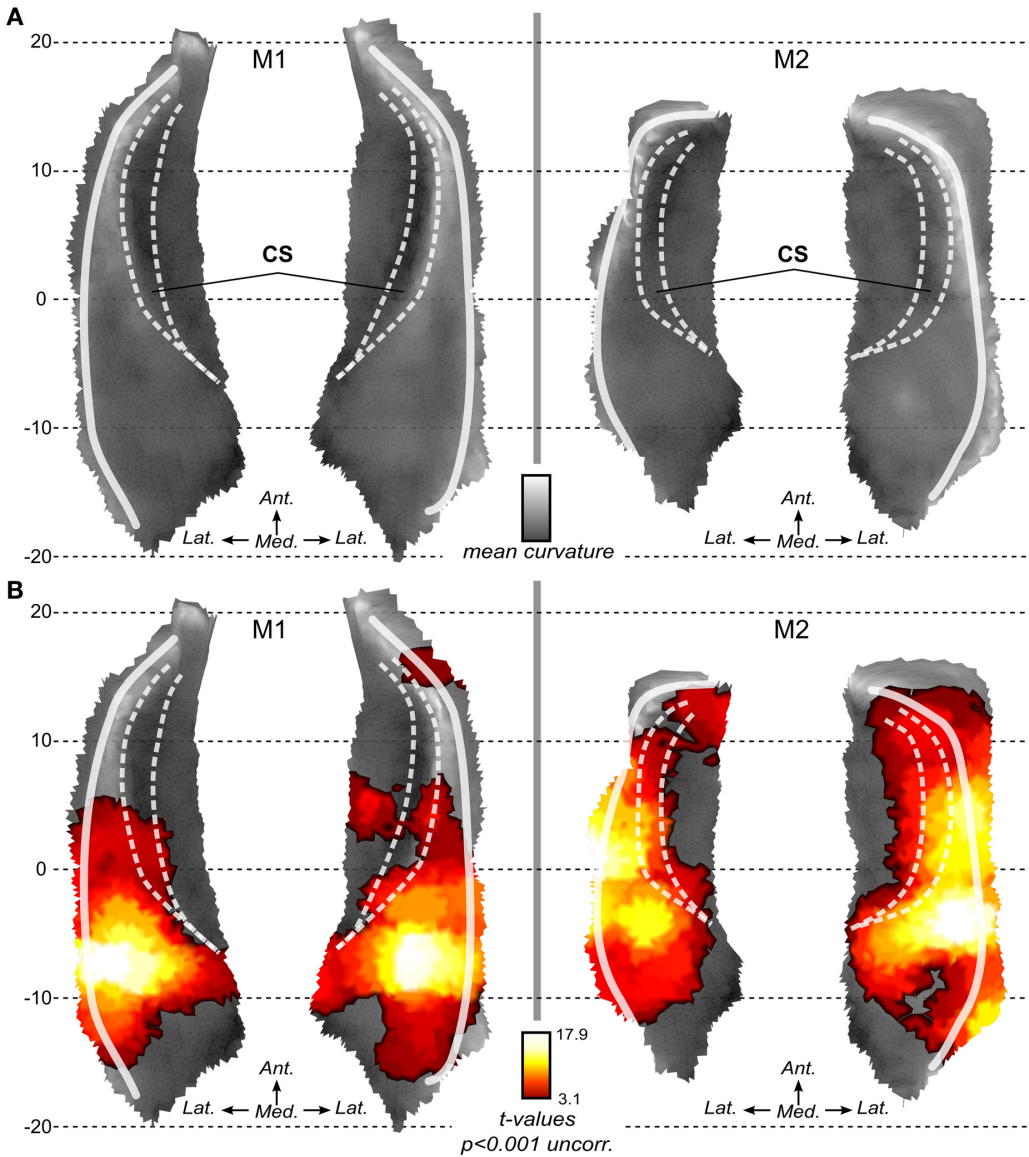
**Local flat maps and SPM maps for sound stimulation. (A)** Local flattening allows the 2D representation of the 3D surface with minimal distortion as compared to full brain flattening. Mean curvature illustrates the original folding of the left and right STP in both monkeys. White lines represent main folding. CS: Circular sulcus is indicated with dashed white lines. **(B)** Activations to sounds as compared to silent baseline. SPM t-maps (volumes) are projected onto the surfaces. Statistical maps are thresholded at *p* < 0.001 uncorrected for multiple comparison.

In both monkeys, we first analyzed the BOLD activation associated with sound stimulation in voxel space. The resulting SPM maps were projected onto the 3D surfaces and maps as shown in Figure 3B. In both animals, activation to sounds was found in most of the superior temporal plane. Robust sound-related activation was found in both monkeys. SPM maps show a rather symmetrical pattern of activation in both subjects. Maxima were found in a region posterior and lateral to the fundus of the circular sulcus illustrated with the medial white line (Figure 3B). Regions with less or no significant activation were found in the most posterior (around putative area Tpt) and most anterior part (temporal pole) of the superior temporal plane and also medial to the fundus of the circular sulcus in the insula.

### Surface-based analyses and frequency preferences

**Figure 4:**
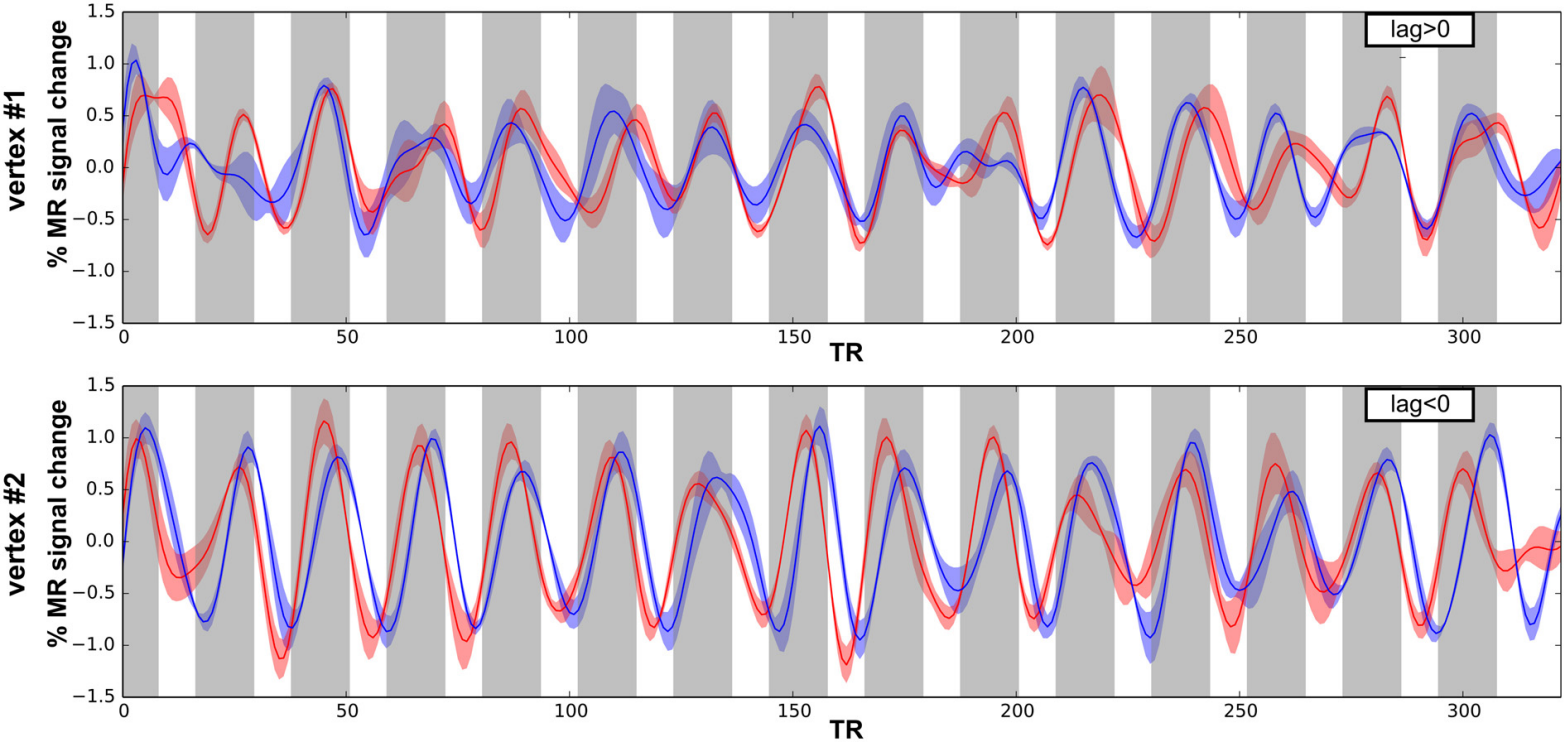
**Times series and cross-correlation.** Average times series for both run types (low-to-high in red; high-to-low in blue), error shadows represent the s.e.m. (across 4 runs) shown for 2 vertices in M1 with either positive (**top**) and negative (**bottom**) lag of maximum cross-correlation.

**Figure 5:**
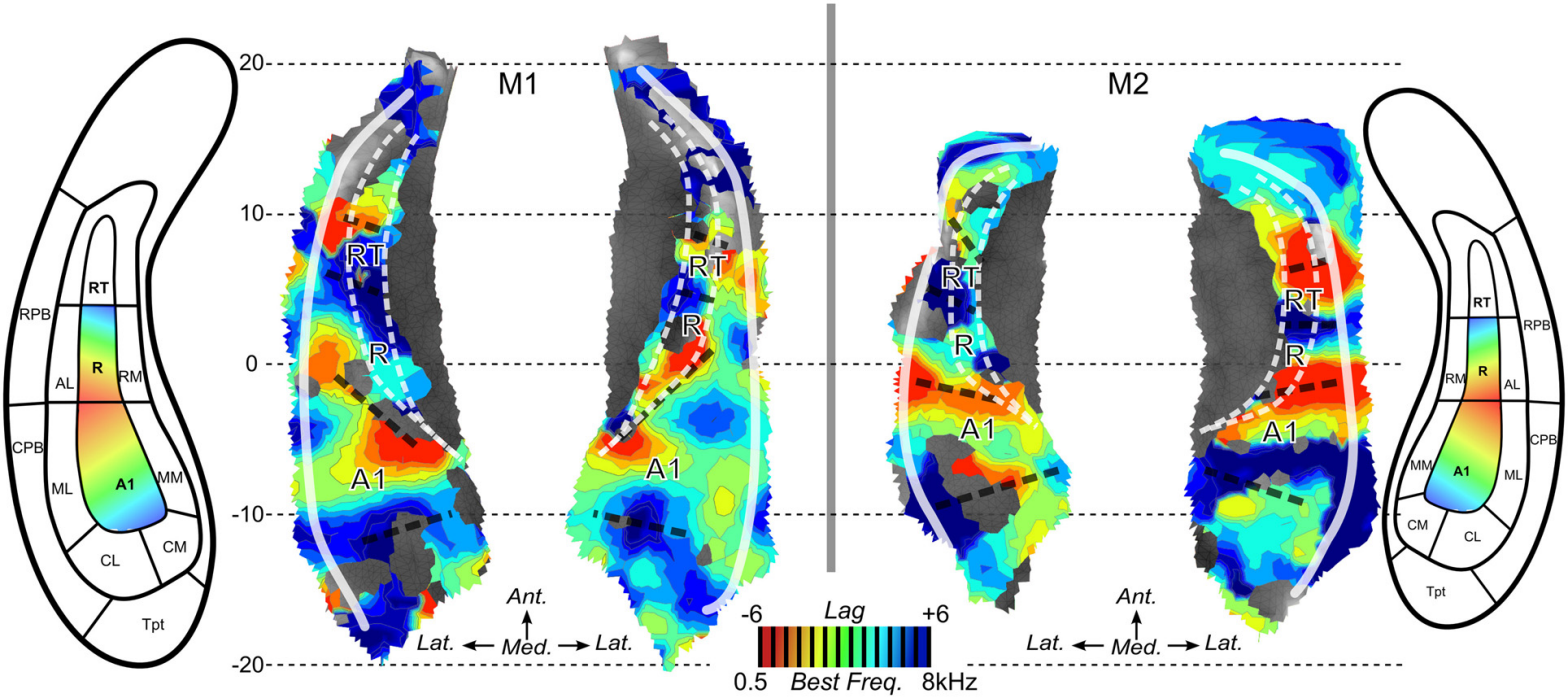
**Best frequency maps.** Maps of best frequency/lag for maximized cross-correlation across run types in M1 and M2. Iso-frequency contours are also mapped. Aligned anatomical atlases are also shown to guide the interpretation of the maps. Dashed black lines illustrate the putative anatomical borders located at the reversals of frequency preference.

Next, we performed surface-based analyses where functional times-series are defined at each point (vertex) of the surface of the superior temporal plane. Time-series for each run-type were averaged, as illustrated in Figure 4, and cross-correlations were computed between the two averaged time-series. Best frequency maps (Figure 5) represent at each vertex the lag with the highest correlation (see Materials and Methods section). Iso-frequency lines were also generated and overlaid to highlight the feature of interest. In both monkeys, largely symmetrical maps were observed. Low-frequency preference regions were found in the 4 hemispheres at the coordinates *y* ~ −3 mm and this region corresponds functionally to the putative border between A1 and R. In both monkeys, this low frequency region was found at the posterior end of the circular sulcus. Posterior to this region, a high frequency preference was often reported at the coordinate *y* ~ −10. This high frequency region, which was less clear in left hemisphere of M2, was located at about 10 mm from the posterior end of the sulcus. In the left hemisphere of M2, the posterior border of A1 with high frequency preference in the medial and lateral part also shows vertices with a low frequency preference and vertices with a very low cross-correlation value in the masked region (gray). The posterior high-frequency region represents a functional putative border between A1 and caudal belt regions (CM and CL). Posterior to A1, nearly 10 mm of cortical surface, according to the adjusted atlas would remain to house the caudal belt regions and area Tpt. In both monkeys, another high frequency region was observed at *y* ~ +5 mm and represents a putative border between area R and area RT. Finally anteriorly, a last low frequency region was observed in all hemispheres but in a lesser extent in the left hemisphere of M2. Co-registered F99 atlas (affine transformation) illustrates the subdivisions of the auditory cortex and highlights the main tonotopic progression in area A1 and R.

### Anatomical features and T1w over T2w ratio

**Figure 6:**
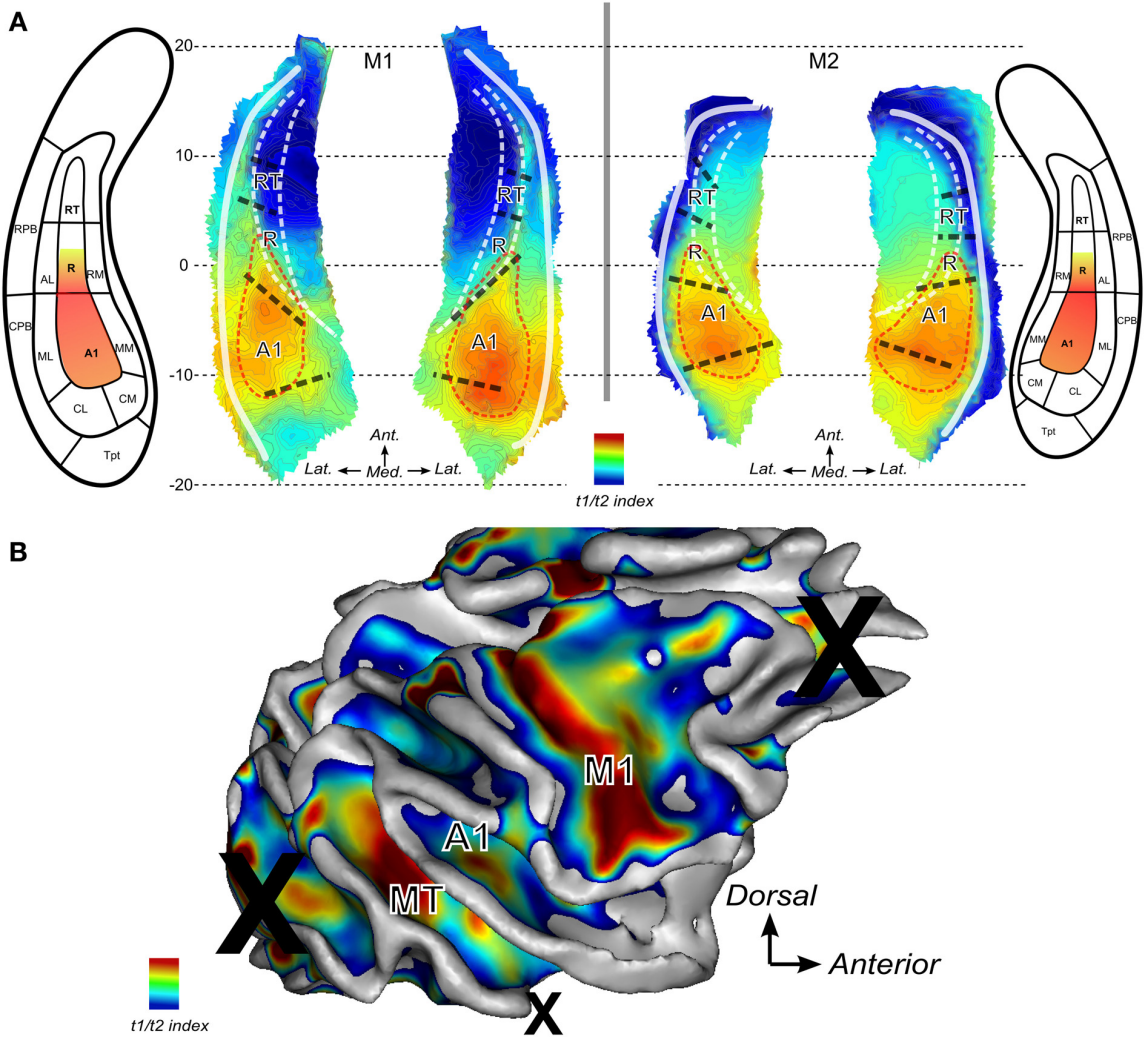
**Anatomical index derived from ratio between T1 and T2 weighted MR images.** The ratio of T1 over T2 weighted MR images is used to derive a gradient of change across cortical thickness. Maps show higher indices where higher intensities are found in gray matter. **(A)** Maps the lateral sulci in M1 and M2. Red dashed lines represent putative auditory core borders. **(B)** Map for the right hemisphere of monkey M2. Several regions show high values: around the primary motor cortex in the central sulcus (label M1), area MT within the STS and in the auditory core (labeled A1).

Finally, the T1w over T2w ratio image was used to compute an index which represents the average intensities across the cortical thickness. For the lateral sulcus, the derived maps are shown in Figure 6A for both monkeys. The Tentative outlines drawn (red dashed lines) and illustrate the cortical region within which auditory core areas A1 and posterior part of R would be located based on this mapping. Highest intensities of gray matter voxels in the ratio T1w over T2w MR image were found within a posterior region of the lateral sulcus where A1 is to be expected according to the anatomical atlas and according to the tonotopic progression in each animal (see Figure 5). High values of this T1/T2 derived index were also found anteriorly where the core region R is predicted from atlas-based parcellation (Figure 6) and from the frequency progression (low-to-high) illustrated in this region (Figure 5). Note that in 3 out of 4 hemispheres (M1:right and M2:left and right), high values were also found to extend posteriorly into caudal belt regions (areas CM and CL).

## Discussion

Here we combine information from functional MRI and anatomical MRI which allows improved parcellation of auditory cortex, and thus improved mapping of function to auditory subfields. We used for the first time in monkeys a phase-encoded fMRI design to map frequency preference in the monkey auditory cortex. Phase-encoded fMRI design has been successfully applied to retinotopic mapping in humans (e.g., Sereno et al., 1995) and in monkeys (Kolster et al., 2009) and to human tonotopic mapping (Talavage et al., 2004; Da Costa et al., 2011; Striem-Amit et al., 2011). Combined with macro-anatomical features (cortical folding) and co-registered surface-based atlas, we present here a detailed tonotopic map of the monkey auditory cortex in good agreement with the current model of organization of the monkey auditory cortex.

### Relation to previous tonotopic mapping in monkeys

Previous tonotopic mappings in monkeys were performed and illustrated in voxel-space, and it is therefore difficult to compare our results with these studies. Previous work (Petkov et al., 2006) used voxel-based representation of oblique fMRI slices (2 mm; averaged 1–3 slices—up to 6 mm) in contrast to our surface mapping. Moreover, the underlying structural images in their study had limited contrast which further complicates the exact assignment to different cortical areas. Tanji et al. (2010) used maximum projection maps to illustrate more precisely the average of signal or *t*-values taken across 4 slices (6 mm) but it remains difficult to relate their map to the flattened representation from Hackett et al. (1998, 2001). In these previous fMRI studies, the circular sulcus is represented with a single line, while a more precise surface representation can illustrate the fundus or floor and the outer bank of the ventral circular sulcus. While A1 mainly lies in the posterior part of the ventral bank of the lateral sulcus which is rather planar, a substantial part of R and RT are found within the circular sulcus and therefore these regions suffer seriously from the axial (voxel-based) representation. This was partly addressed by Tanji et al. (2010) where area RT is shown within the circular sulcus via coronal sections (see their Figure 5) and R stretches from the STP into the adjacent postero-lateral bank of the circular sulcus. To overcome this limitation, 3D surface mapping and isotropic sampling is needed to faithfully represent auditory cortex beyond A1. One further challenge in subdividing the auditory cortex resides in the fact that core and the adjacent lateral belt region share similar frequency preference, that medial belt regions are narrow and therefore difficult to isolate from neighboring core regions with functional MRI. In macaque monkeys, auditory fMRI mapping is more difficult than visual mapping because of the relatively small surface area of the auditory core (A1, R and RT) which is about 100 mm^2^ when V1 is about 1400 mm^2^ (V2 ~ 1000 mm^2^). In the future, our combination of anatomical and functional features could enter a hierarchical clustering algorithm such as Ward's approach that can use the graph connectedness of our triangulated surface and perform an automatic parcellation of the auditory subfields for a given number of clusters.

In our two monkeys, we found a symmetrically bilateral low frequency preference at the posterior end of the circular sulcus which according to co-registered atlas would be the border between A1 and R. Very interestingly, this correspondence between the low frequency preference and a macro-anatomical landmark (posterior part of the circular sulcus) is reminiscent of the recent finding in humans (Da Costa et al., 2011) where low frequency preference was found on the crown of Heshl's gyrus (HG) (or within the sulcal divide of duplicated HG) in 10 subjects. Interestingly, this correspondence between the function and the macro-anatomy is probably the very reason for the success of human tonotopic fMRI (group) studies (e.g., Langers and van Dijk, 2012) which rely on non-rigid normalization into a common brain space (e.g., MNI space). Indeed, these human studies successfully demonstrated the tonotopic organization for at least two reasons: (1) The non-rigid normalization process aligns well the individual Heshl's gyri to the averaged structure of the MNI template. (2) A sufficiently strong correspondence between the tonotopic organization and the underlying macro-anatomy. Moreover, the macro-anatomy in both species suggests that the postero-lateral bank of the circular sulcus in monkeys might roughly correspond to the antero-medial bank of the HG in humans (Baumann et al., 2013). There is often a forme fruste of Heschl's Gyrus in the macaque in the form of a ridge in similar position and orientation relative to the STP (Baumann et al., 2013)

Future developments in voxel-based quantification (VBQ) using quantitative MRI (Sigalovsky et al., 2006; Bock et al., 2013; Weiskopf et al., 2013) would allow the same definition of brain regions in humans and non-human primates. In the current study, we derived a map of cortical myelin and we found highly myelinated regions which seem to be centered into A1, extending anteriorly into posterior part of area R as expected from Hackett et al. (1998). In their anatomical observations, Hackett et al. (1998) illustrated (cf. their Figure 2C) a strong gradient between a very heavily myelinated area A1 toward a much less stained area R and RT: These observations are also in agreement with our maps. However, high values in our maps also extend posteriorly into caudal belt regions. It could be related to recent observations showing involvement in fast processing of sounds and short latencies of neural responses (shorter than in A1) in dorsal auditory regions (Kusmierek and Rauschecker, 2014). Despite its general agreement with known myelin maps (e.g., primary cortices, area MT) it remains difficult to know how our current implementation of cortical myelin mapping is a reliable predictor of core versus belt regions. In the future, this could be assessed in individual monkeys using a co-registration of MRI and post-mortem histological studies. However, in the meantime, improvement in functional MRI resolution and reduction of geometric deformations would be highly beneficial to increase the accuracy in association of functional MRI findings with the different auditory subfields. Before these improvements can be achieved, combining information from anatomical and functional properties, as described here, can help better localize recording and activation sites within the auditory subfields. This will help to guide precisely and efficiently electrophysiological recordings in monkeys and also to relate monkey fMRI results to similar fMRI studies in humans (Da Costa et al., 2011; Dick et al., 2012; Joly et al., 2012a).

### Conflict of interest statement

The authors declare that the research was conducted in the absence of any commercial or financial relationships that could be construed as a potential conflict of interest.

## References

[B1] Baumann S. Griffiths T. D. Rees A. Hunter D. Sun L. Thiele A. (2010). Characterisation of the BOLD response time course at different levels of the auditory pathway in non-human primates. Neuroimage 50, 1099–1108 10.1016/j.neuroimage.2009.12.103 20053384 PMC2880247

[B2] Baumann S. Petkov C. I. Griffiths T. D. (2013). A unified framework for the organization of the primate auditory cortex. Front. Syst. Neurosci. 7:11 10.3389/fnsys.2013.00011 23641203 PMC3639404

[B3] Bock N. A. Hashim E. Janik R. Konyer N. B. Weiss M. Stanisz G. J. (2013). Optimizing T1-weighted imaging of cortical myelin content at 3.0 T. Neuroimage 65, 1–12 10.1016/j.neuroimage.2012.09.051 23036446

[B4] Camalier C. R. D'Angelo W. R. Sterbing-D'Angelo S. J. de la Mothe L. A. Hackett T. A. (2012). Neural latencies across auditory cortex of macaque support a dorsal stream supramodal timing advantage in primates. Proc. Natl. Acad. Sci. U.S.A. 109, 18168–18173 10.1073/pnas.1206387109 23074251 PMC3497796

[B5] Da Costa S. van der Zwaag W. Marques J. P. Frackowiak R. S. J. Clarke S. Saenz M. (2011). Human primary auditory cortex follows the shape of Heschl's gyrus. J. Neurosci. 31, 14067–14075 10.1523/JNEUROSCI.2000-11.2011 21976491 PMC6623669

[B6] Dick F. Tierney A. T. Lutti A. Josephs O. Sereno M. I. Weiskopf N. (2012). *In vivo* functional and myeloarchitectonic mapping of human primary auditory areas. J. Neurosci. 32, 16095–16105 10.1523/JNEUROSCI.1712-12.2012 23152594 PMC3531973

[B7] Engel S. A. (2012). The development and use of phase-encoded functional MRI designs. Neuroimage 62, 1195–1200 10.1016/j.neuroimage.2011.09.059 21985909

[B8] Glasser M. F. Van Essen D. C. (2011). Mapping human cortical areas *in vivo* based on myelin content as revealed by T1- and T2-weighted MRI. J. Neurosci. 31, 11597–11616 10.1523/JNEUROSCI.2180-11.2011 21832190 PMC3167149

[B9] Gruetter R. (1993). Automatic, localized *in vivo* adjustment of all first- and second-order shim coils. Magn. Reson. Med. 29, 804–811 10.1002/mrm.1910290613 8350724

[B10] Hackett T. A. (2011). Information flow in the auditory cortical network. Hear. Res. 271, 133–146 10.1016/j.heares.2010.01.011 20116421 PMC3022347

[B11] Hackett T. A. Preuss T. M. Kaas J. H. (2001). Architectonic identification of the core region in auditory cortex of macaques, chimpanzees, and humans. J. Comp. Neurol. 222, 197–222 10.1002/cne.1407 11745645

[B12] Hackett T. A. Stepniewska I. Kaas J. H. (1998). Subdivisions of auditory cortex and ipsilateral cortical connections of the parabelt auditory cortex in macaque monkeys. J. Comp. Neurol. 394, 475–495 10.1002/(SICI)1096-9861(19980518)394:4<475::AID-CNE6>3.0.CO;2-Z 9590556

[B13] Joly O. Pallier C. Ramus F. Pressnitzer D. Vanduffel W. Orban G. A. (2012a). Processing of vocalizations in humans and monkeys: a comparative fMRI study. Neuroimage 62, 1376–1389 10.1016/j.neuroimage.2012.05.070 22659478

[B14] Joly O. Ramus F. Pressnitzer D. Vanduffel W. Orban G. A. (2012b). Interhemispheric differences in auditory processing revealed by fmri in awake rhesus monkeys. Cereb. Cortex 22, 838–853 10.1093/cercor/bhr150 21709178

[B15] Joly O. Vanduffel W. Orban G. A. (2009). The monkey ventral premotor cortex processes 3D shape from disparity. Neuroimage 47, 262–272 10.1016/j.neuroimage.2009.04.043 19376235

[B16] Jones E. G. Dell'Anna M. E. Molinari M. Rausell E. Hashikawa T. (1995). Subdivisions of macaque monkey auditory cortex revealed by calcium-binding protein immunoreactivity. J. Comp. Neurol. 362, 153–170 10.1002/cne.903620202 8576431

[B17] Kaas J. H. Hackett T. A. (1998). Subdivisions of auditory cortex and levels of processing in primates. Audiol. Neurootol. 3, 73–85 10.1159/000013783 9575378

[B18] Kolster H. Mandeville J. B. Arsenault J. T. Ekstrom L. B. Wald L. L. Vanduffel W. (2009). Visual field map clusters in macaque extrastriate visual cortex. J. Neurosci. 29, 7031–7039 10.1523/JNEUROSCI.0518-09.2009 19474330 PMC2749229

[B19] Kusmierek P. Rauschecker J. P. (2014). Selectivity for space and time in early areas of the auditory dorsal stream in the rhesus monkey. J. Neurophysiol. 111, 1671–1685 10.1152/jn.00436.2013 24501260 PMC4035775

[B20] Langers D. R. M. van Dijk P. (2012). Mapping the tonotopic organization in human auditory cortex with minimally salient acoustic stimulation. Cereb. Cortex 22, 2024–2038 10.1093/cercor/bhr282 21980020 PMC3412441

[B21] Markov N. T. Misery P. Falchier A. Lamy C. Vezoli J. Quilodran R. (2011). Weight consistency specifies regularities of macaque cortical networks. Cereb. Cortex 21, 1254–1272 10.1093/cercor/bhq201 21045004 PMC3097985

[B22] Merzenich M. M. Brugge J. F. (1973). Representation of the cochlear partition of the superior temporal plane of the macaque monkey. Brain Res. 50, 275–296 10.1016/0006-8993(73)90731-2 4196192

[B23] Moerel M. De Martino F. Formisano E. (2012). Processing of natural sounds in human auditory cortex: tonotopy, spectral tuning, and relation to voice sensitivity. J. Neurosci. 32, 14205–14216 10.1523/JNEUROSCI.1388-12.2012 23055490 PMC6622378

[B24] Morel A. Garraghty P. E. Kaas J. H. (1993). Tonotopic organization, architectonic fields, and connections of auditory cortex in macaque monkeys. J. Comp. Neurol. 335, 437–459 10.1002/cne.903350312 7693772

[B25] Morosan P. Rademacher J. Schleicher A. Amunts K. Schormann T. Zilles K. (2001). Human primary auditory cortex: cytoarchitectonic subdivisions and mapping into a spatial reference system. Neuroimage 13, 684–701 10.1006/nimg.2000.0715 11305897

[B26] Peirce J. W. (2007). PsychoPy—psychophysics software in python. J. Neurosci. Methods 162, 8–13 10.1016/j.jneumeth.2006.11.017 17254636 PMC2018741

[B27] Petkov C. I. Kayser C. Augath M. Logothetis N. K. (2006). Functional imaging reveals numerous fields in the monkey auditory cortex. PLoS Biol. 4:e215 10.1371/journal.pbio.0040215 16774452 PMC1479693

[B28] Petkov C. I. Kayser C. Steudel T. Whittingstall K. Augath M. Logothetis N. K. (2008). A voice region in the monkey brain. Nat. Neurosci. 11, 367–374 10.1038/nn2043 18264095

[B29] Rauschecker J. P. Tian B. (2004). Processing of band-passed noise in the lateral auditory belt cortex of the rhesus monkey. J. Neurophysiol. 91, 2578–2589 10.1152/jn.00834.2003 15136602

[B30] Recanzone G. H. (2000). Response profiles of auditory cortical neurons to tones and noise in behaving macaque monkeys. Hear. Res. 150, 104–118 10.1016/S0378-5955(00)00194-5 11077196

[B31] Sereno M. I. Dale A. M. Reppas J. B. Kwong K. K. Belliveau J. W. Brady T. J. (1995). Borders of multiple visual areas in humans revealed by functional magnetic resonance imaging. Science 268, 889–893 10.1126/science.7754376 7754376

[B32] Sigalovsky I. S. Fischl B. Melcher J. R. (2006). Mapping an intrinsic MR property of gray matter in auditory cortex of living humans: a possible marker for primary cortex and hemispheric differences. Neuroimage 32, 1524–1537 10.1016/j.neuroimage.2006.05.023 16806989 PMC1839042

[B33] Striem-Amit E. Hertz U. Amedi A. (2011). Extensive cochleotopic mapping of human auditory cortical fields obtained with phase-encoding FMRI. PLoS ONE 6:e17832 10.1371/journal.pone.0017832 21448274 PMC3063163

[B34] Talavage T. M. Sereno M. I. Melcher J. R. Ledden P. J. Rosen B. R. Dale A. M. (2004). Tonotopic organization in human auditory cortex revealed by progressions of frequency sensitivity. J. Neurophysiol. 91, 1282–1296 10.1152/jn.01125.2002 14614108

[B35] Tanji K. Leopold D. A. Ye F. Q. Zhu C. Malloy M. Saunders R. C. (2010). Effect of sound intensity on tonotopic fMRI maps in the unanesthetized monkey. Neuroimage 49, 150–157 10.1016/j.neuroimage.2009.07.029 19631273 PMC3411355

[B36] Thiele A. Delicato L. S. Roberts M. J. Gieselmann M. A. (2006). A novel electrode-pipette design for simultaneous recording of extracellular spikes and iontophoretic drug application in awake behaving monkeys. J. Neurosci. Methods 158, 207–211 10.1016/j.jneumeth.2006.05.032 16843532 PMC2666830

[B37] Van Essen D. C. Drury H. A. Dickson J. Harwell J. Hanlon D. Anderson C. H. (2001). An integrated software suite for surface-based analyses of cerebral cortex. J. Am. Med. Inform. Assoc. 8, 443–459 10.1136/jamia.2001.0080443 11522765 PMC131042

[B38] Weiskopf N. Suckling J. Williams G. Correia M. M. Inkster B. Tait R. (2013). Quantitative multi-parameter mapping of R1, PD(^*^), MT, and R2(^*^) at 3T: a multi-center validation. Front. Neurosci. 7:95 10.3389/fnins.2013.00095 23772204 PMC3677134

[B39] Yushkevich P. A. Piven J. Hazlett H. C. Smith R. G. Ho S. Gee J. C. (2006). User-guided 3D active contour segmentation of anatomical structures: significantly improved efficiency and reliability. Neuroimage 31, 1116–1128 10.1016/j.neuroimage.2006.01.015 16545965

